# Comparison of retrograde flexible ureteroscopy and percutaneous nephrolithotomy in treating intermediate-size renal stones (2-3cm): a meta-analysis and systematic review

**DOI:** 10.1590/S1677-5538.IBJU.2018.0510

**Published:** 2019

**Authors:** Zhu Zewu, Yu Cui, Zeng Feng, Li Yang, Hequn Chen

**Affiliations:** 1 Department of Urology, Xiangya Hospital, Central South University, Changsha, China

**Keywords:** Ureteroscopy, Nephrolithotomy, Percutaneous, Kidney Calculi

## Abstract

**Purpose:**

To systematically assess the effectiveness and safety of retrograde flexible ureteroscopy (FURS) versus percutaneous nephrolithotomy (PCNL) in treating intermediate-size renal stones (2-3cm).

**Materials and Methods:**

PubMed, Ovid MEDLINE, Web of Science, Cochrane Central Register of Controlled Trials (CENTRAL) and EMBASE were researched to identify relevant studies up to May 2018. Article selection was performed through the search strategy based on Preferred Reporting Items for Systematic Reviews and Meta-Analyses criteria. The Newcastle-Ottawa Scale was applied to assess the methodological quality of case-control studies.

**Results:**

Six retrospective case-controlled trials were included for meta-analysis. The pooled results showed that PCNL was associated with a higher initial stone-free rate (SFR). After more complementary treatments, FURS provided a final SFR (OR: 1.69; 95% CI, 0.93-3.05; P = 0.08) comparable to that achieved by PCNL. PCNL was associated with a higher rate of overall intraoperative complications (OR: 1.48; 95% CI, 1.01-2.17; P = 0.04) and longer hospital stay (MD: 2.21 days; 95% CI, 1.11 to 3.30; P < 0.001). Subgroup analysis by Clavien-graded complication showed PCNL had significantly higher rates of minor complications (OR: 1.58; 95% CI, 1.04-2.41; P = 0.03). No significant difference was noted in major complications (OR: 1.14; 95% CI, 0.53-2.45; P = 0.73) or operative times (MD: −9.71 min; 95% CI, −22.02 to 2.60; P = 0.12).

**Conclusions:**

Multisession FURS is an effective and safe alternative to PCNL for the management of intermediate-size renal stones (2-3cm). It is advisable to balance the benefits and risks according to the individual characteristics of patients and to decide with patients by discussing the advantages and disadvantages of each procedure.

## INTRODUCTION

Currently, percutaneous nephrolithotomy (PCNL) is recommended as the first-line treatment of choice for renal stones more than 2cm in diameter. Due to its high efficiency (1), it continues to have non-negligible morbidity effects such as bleeding requiring angio-embolization, urinoma and organ injury, although rare (2, 3). With the technological advances in flexible ureteroscopy (FURS), coupled with the development of laser lithotripsy systems and novel endoscopic baskets, FURS allows urologists to deal with lower calix stones or even complex renal stones through the natural orifice and achieve an acceptable stone-free rate (SFR) (4). There may be a subset of patients with intermediate-size renal stones (2-3cm) who are amenable to retrograde FURS, whether or not PCNL is contraindicated. Several studies have reported the comparison of PCNL and FURS to manage 2-3cm renal stones, but inconsistences in findings across individual controlled studies have raised concerns as to whether FURS provides a stone-free rate (SFR) comparable to that achieved by PCNL and with fewer complications. Hence, we performed the first meta-analysis to assess the effectiveness and safety of FURS versus PCNL in the treatment of 2-3cm renal stones.

## MATERIALS AND METHODS

### Data Sources and Search

We searched PubMed, Ovid MEDLINE, Web of Science, Cochrane Central Register of Controlled Trials (CENTRAL) and EMBASE to identify relevant studies up to May 2018. The search strategy was done by two authors independently with the following search terms: (percutaneous nephrolithotomy OR percutaneous lithotripsy OR micropercutaneous nephrolithotomy OR PCNL OR mini-PCNL) AND (retrograde intrarenal surgery OR flexible ureteroscopy OR RIRS OR FURS) AND (renal calculi OR renal stones OR nephrolithiasis). A reference list of studies included in the synthesis and analysis was also screened to identify additional reports.

### Study Selection

Article selection was performed independently according to the process based on Preferred Reporting Items for Systematic Reviews and Meta-Analyses (PRISMA) guidelines (5). Inclusion criteria for final selected studies were as follows: (1) language limited to English; (2) renal stones were 2-3cm in diameter, with no location and number restriction; and (3) original comparative studies reporting at least one of the following outcomes of both PCNL and FURS: SFR, treatment session, operation time, overall complications, or Clavien grade complication (6). However, studies fulfilling any of the following exclusion criteria were excluded: (1) inclusion of pediatric patients (< 18 years old) and (2) studies published as conference abstracts or posters.

### Quality Assessment

According to the widely used criteria provided by the Oxford Centre for Evidence-Based Medicine (7), the Newcastle-Ottawa Scale (NOS), with scores ranging from 0 to 9, was applied to assess the methodological quality of case-control studies (8). The items of the NOS for case-control studies contain selection, comparability and exposure. A score of 3 points or less was judged as low quality; scores of 4-5, as moderate quality; and scores of 6 points or more, as high quality. The methodological quality was rated for each included study by two authors independently, and any discrepancies were resolved by discussion or by consulting a third author.

### Data Extraction and Analysis

Data were extracted independently using standard data extraction forms. We did not find repetitive publication of any study during the data abstraction. Odds ratios (ORs) were used for the binary variables, and mean differences (MDs) were used for the continuous parameters and presented as the means and standard deviations if the scales among studies were consistent. Otherwise, the standardized mean difference (SMD) was used. Heterogeneity among studies was identified with the inconsistency (I2) and *χ* ^2^ statistical methods. An I2 value > 50% or P value < 0.10 indicated significant heterogeneity (9). Where heterogeneity among studies was not detected, pooled estimates were calculated with a fixed-effect model (Mantel-Haenszel method) (10). If significant heterogeneity was detected, a random-effect model (DerSimonian-Laird method) (11) was used, and sensitivity analysis was applied to explore the reliability of the results by omitting a specific study each time. A **Z** test was used to determine the pooled effects, and a P value > 0.05 indicated no statistical significance (12). A funnel plot was routinely constructed to evaluate publication bias (13). All data analysis was performed with Review Manager Software (RevMan v.5.3, Cochrane Collaboration, Oxford, UK) and STATA 12.0 (Stata Corp LP, College Station, TX, USA).

## RESULTS

### Search results and Study Characteristics

**Figure 1:**
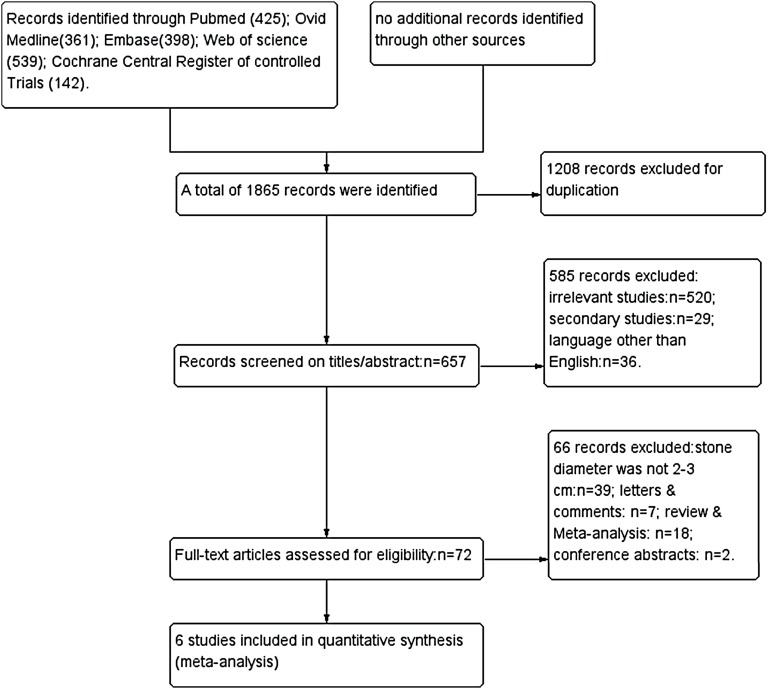
Preferred Reporting Items for Systematic Reviews and Meta-analyses flow of study selection.

As illustrated in Figure 1, 657 studies were initially identified, and a total of 6 retrospective case-controlled trials (rCCTs) were eligible for final inclusion, all of which were full-text articles. We did not find any additional records identified through reference lists. The baseline characteristics of the 6 studies are shown in Table-1. Basic characteristics, such as age, sex ratio, body mass index, stone size and stone location, were described as comparable between the two groups according to each study. Some variation of inclusive criteria among studies was observed, and patients with multiple stones were excluded in 2 studies (14, 15). Furthermore, as for stone location, 2 studies only included renal pelvis stones (16) and lower calyceal stones (15), respectively, and no location restriction was noted in the rest of the included studies. Only 1 study included patients with either functional or anatomical solitary kidneys (15). Inconsistency in the definition of SFR was observed among the included studies, and one study reported that a residual fragment < 4mm in diameter was considered an insignificant clinical fragment (17). The rest of the included studies defined a residual fragment < 2mm as an insignificant clinical fragment. The imaging techniques used to determine the SFR varied and included CT (computer tomography), US (ultrasound) and KUB (X-ray of the kidney, ureter and bladder). The surgical techniques for PCNL varied in terms of image guidance, dilator, sheath size, and lithotripsy technique. Regarding the FURS techniques, the variations among studies were dilator and laser setting (Table-2).

**Table 1:** Basic characteristics of included references.

Reference (year)	Country	SQ	LE	Cases (M/F), n		Mean (SD) age, years		BMI		Stone burden (Mean± SD)		Inclusion criteria	SF station definition
				PCNL	RIRS	PCNL	RIRS	PNCL	RIRS	PNCL	RIRS		
Hyams et al. (2009) (17)	USA	6✯	3b	20(11/9)	19(11/8)	48(-)	56(-)	26.2(-)	30.5(-)	2.4 cm (-)	2.4 cm (-)	single or multiple stone, any location	Residual stone <4 mm with KUB or US or CT
Pan et al. (2013) (14)	China	6✯	3b	74(34/40)	80(38/42)	45.6(-)	48.3(-)	NR	NR	2.6 cm ±0.3	2.3 cm ± 0.4	single stone, any location	Residual stone <2 mm with CT
Zengin et al. (2015) (16)	Turkey	6✯	3b	54(22/32)	54(29/25)	53.5(13.1)	48.7(15.6)	25.7(3.7)	26(5)	25.4 mm ± 4.4	25.1 mm ± 6.6	single or multiple stone, renal pelvic	Residual stone <2 mm with CT
Pieras et al. (2017) (18)	Spain	6✯	3b	59(37/22)	56(36/20)	49.37(14.2)	49.32(13.7)	23.52(3.7)	23.69(3.6)	22.37 mm ± 2.7	22.28 mm ± 2.6	single or multiple stone, any location	Residual stone <4 mm with plain radiography and US
Zhang, et al. (2018) (15)	China	5✯	3b	42(25/14)	34(20/14)	39.7 (1.6)	40.2(1.8)	22.58 (3.62)	23.24(3.18)	25.8 mm ± 3.6	24.1 mm ± 4.5	single stone, solitary kidney (functional or anatomical), lower calyceal	Residual stone <2 mm with CT
Chen et al. (2018) (19)	China	6✯	3b	106(49/57)	148(65/83)	52.1(17.1)	54.3(14.8)	34.7 (6.5)	36.3(7.2)	24.7 mm ± 5.1	25.4 mm ± 4.6	single or multiple stone, any location	Residual stone <2 mm with KUB and US or CT

✯ Retrospective case-controlled trials were assessed with the Newcastle-Ottawa Scale;

**BMI** = body mass index; **CT** = computer tomography; **F** = female; **KUB** = X-ray of the kidney, ureter and bladder; **LE** = level of evidence; **M** = male; **NR** = not reported; **PCNL** = percutaneous nephrolithotomy; **RIRS** = retrograde intrarenal surgery; **SD** = standard deviation; **SF** = stone free; **SQ** = study quality; and **US** = ultrasound.

**Table 2:** An overview of techniques applied in PCNL and RIRS.

Study	PCNL technique					RIRS technique				
	Imaging	Dilator	Sheath size	Lithotripsy technique	Nephrostomy tube	Dilator	UAS	Ureteroscope	Laser setting (fiber, energy, frequency, power)	DJ stent
Hyams et al. (2009) (17)	X-ray	Balloon	NR	Pneumatic/ Ultrasonic/Laser	R	Balloon (S)	S	flexible	NR	R
Pan et al. (2013) (14)	X-ray	Amplatz	18F	NR	R	Rigid ureteroscopy	12F (S)	5.3F/6.9F, flexible	0.8-1.2 J, 8–10 Hz	R
Zengin et al. (2015) (16)	X-ray	Amplatz	30F	Pneumatic	R	Rigid ureteroscopy	11/13F (R)	7.5F, flexible	NR	NR
Pieras et al. (2017) (18)	US	Balloon/Metal	24F	NR	R	Semirigid ureteroscopy	11/13/15F (R)	flexible	270 µm, 0.4-0.8 J, 800-1200 Hz	R
Zhang et al. (2018) (15)	X-ray/US	Amplatz	16/18F	Laser	R	Semirigid ureteroscopy	14 F (R)	7.5F, flexible	200 µm, 20 W, 0.6-1.0 J, 10-20 Hz	R
Chen et al. (2018) (19)	X-ray	Amplatz	18/20F	Laser	R	Semirigid ureteroscopy	12F	flexible	200 μm, 12–20 W, 14–20 Hz	R

**DJ** = double J ureteral stent; **NR** = not reported; **R** = routine; **S** = selective; **UAS** = ureteral access sheath; **US** = ultrasound

### Study quality

According to the Newcastle-Ottawa Scale (NOS), the methodological quality of the six rCCTs was judged to be high (LE 3b; NOS: 6 of 9 points) (14, 16-19) and medium for 1 rCCT (LE 3b; NOS: 5 of 9 points) (15). The risk of bias assessment for each included rCCT is illustrated in Table-3.

**Table 3:** Risk of bias assessment for included retrospective controlled trials.

Study	Selection				Comparability		Exposure			Scores
	a	b	c	d	e	f	g	h	i	
Hyams et al.(2009)	**★**	**★**			**★**		**★**	**★**	**★**	6
Pan et al.(2013)	**★**	**★**		**★**	**★**		**★**	**★**		6
Zengin et al. (2015)	**★**	**★**		**★**	**★**		**★**	**★**		6
Pieras et al. (2017)	**★**	**★**		**★**	**★**		**★**	**★**		6
Zhang et al. (2018)	**★**	**★**			**★**		**★**	**★**		5
Chen et al. (2018)	**★**	**★**			**★**		**★**	**★**	**★**	6

1 **a** = adequate case definition; **b** = representativeness of the cases; **c** = selection of controls; **d** = definition of controls; **e** = study controls for the most important factor; **f** = study controls for any additional factor; **g** = ascertainment of exposure; **h** = some methods of ascertainment for cases and controls; and **i** = non-response rate

## META-ANALYSIS

### Stone-free rate

**Figure 2:**
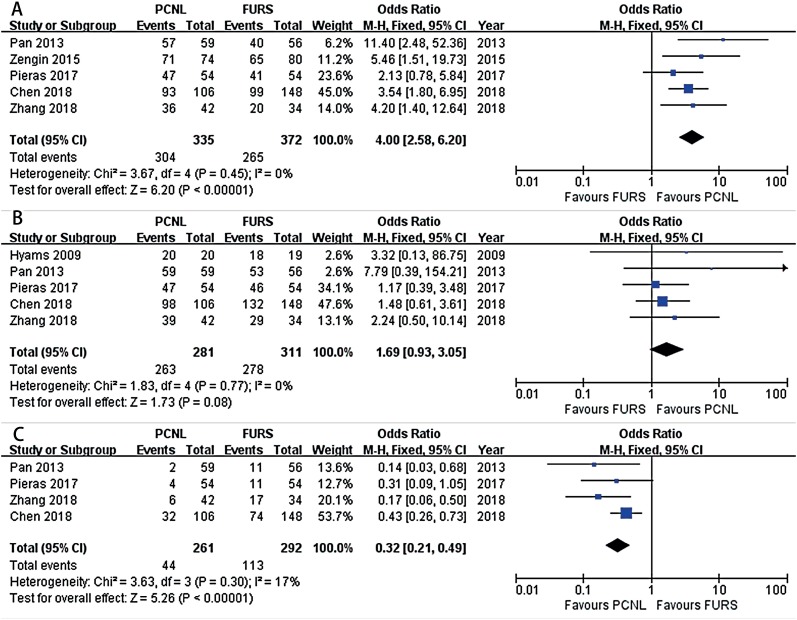
Forest plots illustrating meta-analysis of (A) initial SFR; (B) final SFR; and (C) multiple treatments.

Comparison of the initial SFR between the two groups was reported in five studies (14-16, 18, 19), and the pooled result indicated that compared to FURS, PCNL provided a significantly higher initial SFR (OR: 4.00; 95% CI, 2.58-6.20; P < 0.001, Figure-2A) without detection of statistical heterogeneity (I2 = 0%). In terms of final SFR after multiple sessions, five studies had enough data available for this measure and thus were included in the analysis (14, 15, 17-19). The pooled result showed that multiple-session FURS provided a final SFR comparable with that of PCNL (OR: 1.69; 95% CI, 0.93-3.05; P = 0.08, Figure-2B), without the detection of statistical heterogeneity (I2 = 0%).

### Complementary treatments

Four studies with data available for the combination reported the number of patients needing complementary treatment (14, 15, 18, 19). The meta-analysis showed that the FURS group was associated with significantly more complementary treatments (OR: 0.32; 95% CI, 0.21-0.49; P < 0.001, Figure-2C), with the detection of low statistical heterogeneity (I2 = 17%).

### Complications

**Figure 3:**
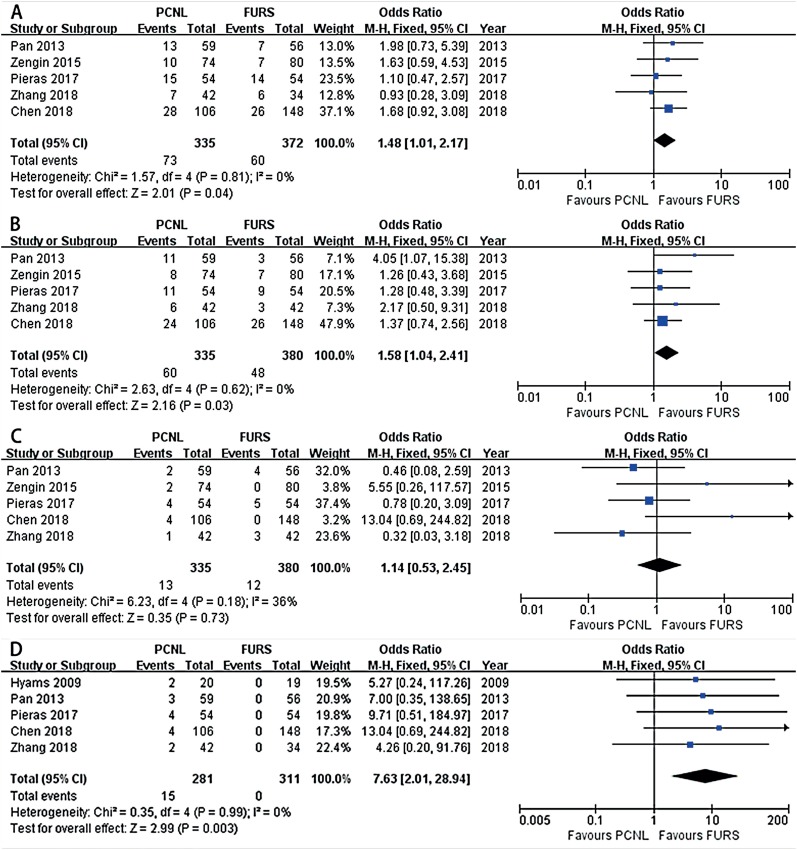
Forest plots illustrating meta-analysis of (A) overall complications; (B) minor complications; (C) major complications; and (D) transfusion rates.

Five included studies had enough data relevant to intraoperative complications (14-16, 18, 19). The pooled results showed that PCNL was associated with a significantly higher rate of overall complications (OR: 1.48; 95% CI, 1.01-2.17; P = 0.04, Figure-3A) without detection of statistical heterogeneity (I2 = 0%). Subgroup analysis by Clavien-graded complication (6) illustrated that PCNL had a significantly higher rate of minor complications (OR: 1.58; 95% CI, 1.04-2.41; P = 0.03, Figure-3B) and no significant difference in major complications (OR: 1.14; 95% CI, 0.53-2.45; P = 0.73, Figure-3C). Regarding each complication, enough data for meta-analysis were only available for bleeding needing transfusion. The pooled results based on five studies showed that PCNL was associated with a significantly higher rate of transfusion (OR: 7.63; 95% CI, 2.01-28.94; P = 0.003, Figure-3D).

### Operative time

**Figure 4:**
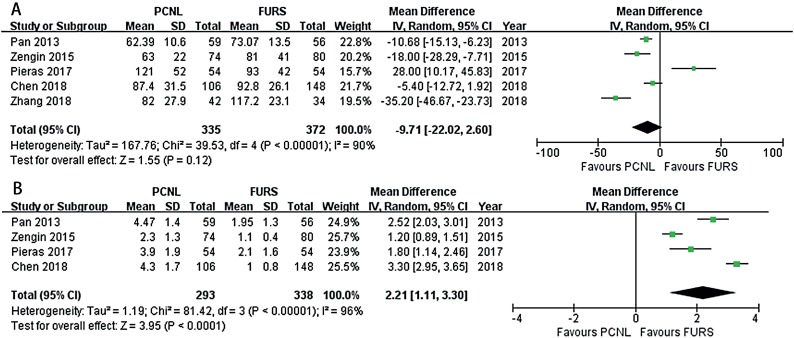
Forest plots illustrating meta-analysis of (A) operative time and (B) hospital stay.

Five included studies provided enough data available on operative times (14-16, 18, 19). High heterogeneity (I2 = 90%) among studies was identified; thus, a random-effect model was used. The pooled results showed no significant difference between the two groups with regard to operative time (MD: −9.71 min; 95% CI, −22.02 to 2.60; P = 0.12, Figure-4A).

### Hospital stay

Four included studies had enough data relevant to hospital stay (14, 16, 18, 19). High heterogeneity (I2 = 96%) among studies was identified, so a random-effect model was used. The result of meta-analysis indicated that PCNL was associated with a significantly longer length of hospital stay (MD: 2.21 days; 95% CI, 1.11 to 3.30; P < 0.001, Figure-4B).

### Publication bias and sensitivity analysis

**Figure 5:**
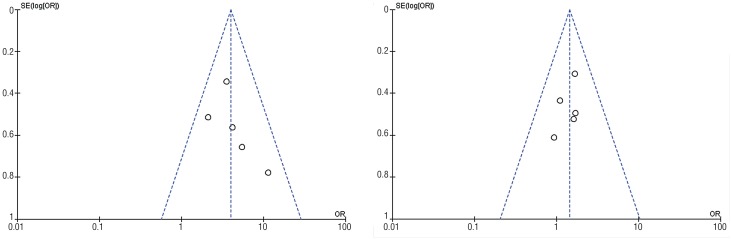
Funnel plot of comparison regarding (A) final SFR and (B) overall complications.

**Figure 6:**
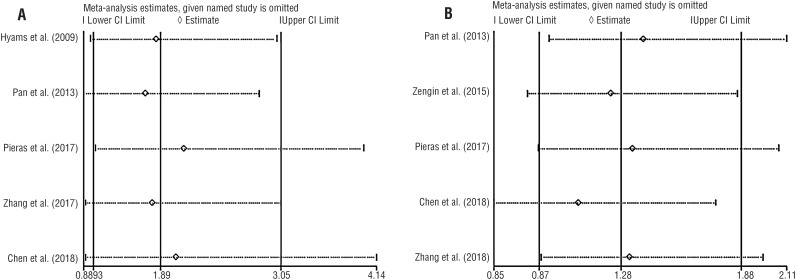
Sensitivity analysis of comparison regarding (A) final SFR and (B) overall complications.

The funnel plots did not show any visual publication bias (Figure-5). The results of sensitivity analysis showed that the outcome of overall complications was unstable, while the others were stable (Figure-6), even though significant heterogeneity was detected in both operative time and hospital stay.

## DISCUSSION

In our current study, the synthesis results showed that PCNL offered an initial SFR superior to retrograde FURS in the management of 2-3cm renal stones, which was consistent with most of the included studies. The potential reasons for one-stage FURS with a relatively lower SFR may be that residual fragments are more likely to represent a cluster of clinically insignificant fragments, in addition to the inherent shortcomings of current FURS techniques and systems, such as limited working channels and the flexibility of ureteroscopy (20). To improve the initial SFR of FURS, Bryniarski et al. introduced the method by changing the position of the patient to relocate lower pole stones (21). Both Mulţescu et al. and Cho et al. recommended that dusting then fragmentation can be better for stones larger than 1cm because the dust may hinder visualization of the clear operative field and the difficulty of differentiating a small fragmented stone in the midst of dust (20, 22). Kuo et al. suggested that small fibers (200-270μm) are superior to larger ones (365μm), because they facilitate flexibility and fluid irrigation without decreasing fragmentation efficacy (23). Chen et al. introduced a novel method to aspirate the fragments directly by vacuum aspiration UAS combined with artificial water circulation created by injecting saline into the tail end of the ureteral catheter (19), which also helped decrease the operative time due to the decreased use of baskets. In the foreseeable future, improvements in laser fibers with higher-energy transport and the combination of higher flexibility and a smaller caliber of endoscopes will probably allow us to greatly improve the SFR of one-session FURS.

Inconsistency in whether FURS provided a final SFR comparable to that achieved by PCNL was noted among the included studies. Zengin et al. reported that the final SFRs were 95.5% in the PCNL group and 80.6% in the FURS group-1 month post operation, while Chen et al. reported that the FURS group was similar to the PCNL group regarding the final SFR (89.1 vs. 92.5%). We may accept the inconsistency with reservation, because a satisfied SFR could be achieved by multisession FURS, which was evidenced by the latest study reporting that the SFR of single-session FURS was 67.2% and the final SFR achieved after multisession procedures was 89.1% (19). This result has been reinforced by similar studies (21, 24, 25). Additionally, the decision for axillary treatment was largely influenced by surgeon preferences and individual patient preferences. In our current meta-analysis, the forest plot showed that FURS offered a final SFR comparable to that achieved by PCNL at the expense of more axillary procedures, indicating that multisession FURS was as an effective alternative choice to PCNL for the management of 2-3cm renal stones. Kang et al. performed a meta-analysis comparing the final SFR between FURS and PCNL in treating renal stones > 2cm in diameter and found that PCNL was superior to FURS (RR: 1.11; 95% CI 1.02-1.21, P < 0.014), with significant heterogeneity (26). The inconsistency may be caused by the included studies with different stone burdens, where a substantial amount of variation in stone diameter ranging from 2cm to 4cm or even larger was noted in the previous meta-analysis of Kang et al., because the stone volume would multiply with the increase in diameter.

Some postoperative complications were unique to each group, and those unique to PCNL were bleeding needing transfusion or even embolization, prolonged urine leaks and pelvic perforation, whereas ureteral injuries and ureteral strictures were unique to FURS. Hence, we compared the Clavien-graded complications (6) and found that PCNL was associated with a significantly higher rate of transfusion, leading to a higher incidence of minor complications and overall complications. The potential risk of bleeding secondary to PCNL should be taken into serious consideration, especially in patients with solitary kidneys. These patients are at a higher risk of acute renal failure when treated with PCNL than patients with bilateral kidneys, because they are more likely to suffer bleeding needing embolization as a consequence of the compensatory hypertrophy (27). Additionally, urinary obstruction by blood clots (28) and functional parenchymal loss after PCNL deteriorate the function of a solitary kidney. Bai et al. observed that FURS was a safe alternative to PCNL in patients with solitary kidneys, reporting that 11.7% (7/60) of patients treated with PCNL encountered bleeding requiring transfusion, but no patient (0/56) in the FURS group required transfusion (29). Additionally, Giusti et al. as well as Shi et al. also confirmed the sufficient safety of FURS in patients with solitary kidneys (28, 30). Regarding the major complications (Clavien grades III and IV), they were closely related to serious bleeding secondary with PCNL, while they were closely related to urosepsis with FURS. The current meta-analysis showed that there were no significant differences in major complications. Theoretically, larger renal stones resulted in a significantly longer operation time, which largely increased the risk of urosepsis secondary to FURS. Without prompt management, sepsis would be dangerous and even life-threatening. Somani et al. analyzed complications associated with ureterorenoscopy based on the CROES database with a total of 11.885 patients and found that one cause of death (1 / 5) was described as sepsis during the 3-month follow-up period (31). Blackmur et al. found that positive urine culture and large stone size were significantly associated with postoperative urosepsis by matched-pair analysis (32). Thus, it is vital to get preoperative microbiological evaluation and limit the surgical time to no more than 90-120 minutes during the FURS procedure (33).

Generally, SFR and complications are evaluated as the primary factors affecting clinical decisions. When we take the efficacy quotient (EQ) into consideration during the decision making, it favors PCNL with a higher EQ due to a similar final SFR and a lower retreatment rate (30). Further, considering the multiple-session FURS with comparable final SFR and lower rates of bleeding as well as shorter recovery time, multisession FURS is an effective and safe alternative to manage intermediate-size renal stones in patients with contraindications to PCNL. Cost and outcome analysis may also help us make decisions for the treatment of moderate renal stones. Pan found that the initial medical expenditure including hospitalization, laboratory and radiology costs favored the FURS group; however, counting the retreatment costs in the two groups, the total medical expenditure was similar between the two groups (14), while Hyams et al. found that the total cost of PCNL was significantly greater than that of FURS when axillary procedures were taken into consideration (17). There are contradictory conclusions between a few reported references due to different medical settings, a variety of surgical techniques and challenges in standardizing costs (14, 17, 34), and further studies of the relative costs of these procedures are needed. Regardless, it is advisable to balance the benefits and risks according to the individual characteristics of patients and to decide with patients by discussing the advantages and disadvantages of each procedure.

The current meta-analysis has several potential limitations. First, all included studies were retrospective studies, and the number of included cases was relatively small, which may have reduced the reliability of the evidence in our meta-analysis. Second, we included published references but excluded conference abstracts to assess the methodology precisely and get detailed data, which may have resulted in potential publication bias, even though the funnel plot showed no significant published bias. Third, the pooled results of overall complications should be interpreted with caution due to the instability detected by sensitivity analysis, and high heterogeneity was noted in several continuous parameters such as operating time and hospital stay, even though the sensitivity analysis showed that the outcomes were stable. The heterogeneity may be attributed to the diversity of surgeon experience, perioperative management, and techniques, especially for PCNL. Lastly, the study was not able to obtain full clinical outcomes for the complementary treatments. With these data being included, it might show different results in terms of intraoperative complications, operative time and hospital stay.

## CONCLUSIONS

PCNL offered a higher initial SFR as well as fewer episodes of retreatment. Multisession FURS could provide a comparable final SFR and shorter recovery time with fewer overall complications in the treatment of intermediate-size renal stones (2-3cm), which could indicate that FURS is an effective and safe alternative to PCNL in the treatment of patients with intermediate-size (2-3cm) renal stones. Therefore, it is advisable to balance benefits and risks according to the characteristics of individuals and choose the optimal option for patients. Nevertheless, further prospective randomized trials are required to confirm the results.
